# The integrity of thalamo-dorsolateral prefrontal cortex tract: a key factor in residual consciousness in disorders of consciousness patients

**DOI:** 10.3389/fneur.2024.1373750

**Published:** 2024-08-14

**Authors:** Ji Yoon Jung, Yeun Jie Yoo, Mi-Jeong Yoon, Bo Young Hong, Tae-Woo Kim, Geun-Young Park, Jong In Lee, Soo-Hwan Lee, Sun Im, Seong Hoon Lim

**Affiliations:** ^1^Department of Rehabilitation Medicine, St. Vincent’s Hospital, College of Medicine, The Catholic University of Korea, Seoul, Republic of Korea; ^2^Department of Rehabilitation Medicine, National Traffic Injury Rehabilitation Hospital, Gyeonggi-do, Republic of Korea; ^3^Department of Rehabilitation Medicine, Bucheon St. Mary’s Hospital, College of Medicine, The Catholic University of Korea, Seoul, Republic of Korea; ^4^Department of Rehabilitation Medicine, Seoul St. Mary’s Hospital, College of Medicine, The Catholic University of Korea, Seoul, Republic of Korea; ^5^Institute for Basic Medical Science, Catholic Medical Center, The Catholic University of Korea, Seoul, Republic of Korea

**Keywords:** consciousness disorders, minimally conscious state, thalamus, dorsolateral prefrontal cortex, diffusion tensor imaging, prognosis

## Abstract

**Background:**

The mesocircuit model describes a complex network that includes the prefrontal cortical-striatopallidal-thalamo-cortical loop systems and is involved in the mechanism underlying consciousness in patients with disorders of consciousness (DoC). Inhibitory signals to the thalamus become hyperactive in DoC patients, leading to a loss of consciousness. Reactivating this mesocircuit system is important for recovering consciousness in these patients. We investigated how the residual integrity of the thalamo-dorsolateral prefrontal cortex tract (TDLPFCT) influences consciousness in patients with DoC.

**Methods:**

This retrospective case–control study included three groups: prolonged DoC (*n* = 20), stroke without DoC (*n* = 20), and healthy controls (*n* = 20). Diffusion tensor imaging (DTI) was performed at least 4 weeks after the onset. Thalamo-DLPFC tracts were reconstructed using diffusion tensor tractography, and fractional anisotropy (FA) and tract volume (TV) were measured for each hemisphere. Consciousness was assessed using the revised coma recovery scale (CRS-R) within a week of brain imaging.

**Results:**

Significant differences in DLPFCT TV were observed across all three groups, in both affected and less-affected lobes, with the DoC group showing the greatest reduction. A significant correlation was found between the TV of the less-affected TDLPFCT and CRS-R score.

**Conclusion:**

The integrity of the TDLPFCT, particularly in the less affected hemisphere, is associated with consciousness levels in patients with prolonged DoC. This finding suggests its potential importance in assessing prognosis and further developing therapeutic strategies for patients with DoC.

## Introduction

Disorders of consciousness (DoC) typically arise from several brain disorders, such as stroke, traumatic brain injury, or hypoxic brain injury, leading to changes in arousal and/or awareness (1). DoC encompasses conditions such as coma (2), unresponsive wakefulness syndrome (UWS) (3), and minimally conscious state (MCS) (4). Coma is a state induced by severe brain injury, where patients are unable to awaken and are not aware of themselves or their surroundings (5). Unresponsive wakefulness syndrome (UWS), previously termed vegetative state (VS), is defined by the complete lack of behavioral evidence indicating awareness of self or the environment (3). Unlike UWS, the minimally conscious state (MCS) involves a partial retention of conscious awareness. Patients in MCS display detectable signs of consciousness, but these behaviors are not consistently reproducible (4). Given the complex mechanisms and etiology of DoC, precisely predicting the prognosis of DoC patients poses a significant challenge to date (1).

The “mesocircuit model,” a complex network and signaling system involving the prefrontal cortical-striatopallidal-thalamo-cortical loop systems may contribute to consciousness (6). In patients with DoC, inhibitory signals to the thalamus might become hyperactive, leading to a loss of consciousness (7). Thus, reactivation of the mesocircuit system may be crucial for regaining consciousness in these patients.

Recent studies have shown that the connectivity between the thalamus and the prefrontal cortex significantly influences consciousness levels in DoC patients. It has been found that the integrity of thalamo-prefrontal connectivity is significantly associated with the ability of traumatic brain injury (TBI) patients to follow commands at an early stage (8).

Some studies have particularly emphasized the role of the dorsolateral prefrontal cortex (DLPFC), a subregion of the PFC known for its crucial role in working memory (9–11), and as part of the executive control network of the frontoparietal network, which mediates attention and environmental awareness (12). The role of the DLPFC in maintaining prolonged alertness and focus has been associated with its relevance to patients diagnosed with DoC (13). A previous study demonstrated that short-term anodal transcranial direct current stimulation (tDCS) of the left DLPFC transiently improved Coma Recovery Scale-Revised (CRS-R) scores in MCS patients (14, 15).

As previously mentioned, the mesocircuit model centers on the involvement of central thalamic neurons and their connections to the frontostriatal system. The central thalamus, including the intralaminar and associated paralaminar portions of related thalamic association nuclei including mediodorsal thalamus, is typically the primary area of involvement in DoC (16). The mediodorsal thalamus, as a part of the central thalamus, is known as the subregion that sends the largest output to the prefrontal cortex (8, 9).

Thus, assessing the activity or integrity of the connectivity between the MD thalamus and the DLPFC could present a novel approach for evaluating residual consciousness in DoC patients. Our hypothesis posits that the relatively preserved thalamo-dorsolateral prefrontal cortex tract (TDLPFCT) is a significant factor in determining consciousness levels in patients with prolonged DoC. Furthermore, damage to the integrity of the thalamo-prefrontal tract may be a contributing factor to DoC. Consequently, we designed our study to investigate the potential correlation between the integrity of TDLPFCT and residual consciousness in patients with prolonged DoC, and we aimed to apply these findings in actual clinical practice. Furthermore, we sought to compare the integrity of TDLPFCT in individuals with prolonged DoC, those who have experienced stroke with preserved awakeness, and a healthy control group.

## Materials and methods

### Subjects and study design

This study was a retrospective case–control study. A total of 60 subjects were assigned into three groups (20 participants in each group respectively): (1) Prolonged disorders of consciousness (DoC) group, (*n* = 20); (2) stroke without DoC group (*n* = 20); and (3) healthy control group (*n* = 20). Prolonged DoC is defined as “any state of altered consciousness resulting from a sudden onset brain injury that persists for at least 4 weeks” (17). Participants in the stroke without DoC group, and 12 participants of DoC group were retrospectively selected from the Department of Rehabilitation medicine, St. Vincent’s Hospital (Suwon, South Korea), while eight participants of DoC group were recruited from the Department of Rehabilitation medicine, Bucheon St. Mary’s Hospital (Bucheon, South Korea), between October, 2019 and September, 2022.

The consciousness level of DoC participants was evaluated with revised coma recovery scale (CRS-R), which is commonly used to evaluate the status and prognosis of DoC patients (18, 19). All DoC group patients met the following inclusion criteria: (1) adult patients over 20 years of age who have experienced prolonged disorders of consciousness for at least 4 weeks due to stroke, traumatic brain injury, or ischemic brain injury; (2) patients who have undergone 3.0-T magnetic resonance imaging of brain (brain MRI) scan and diffusion tensor imaging (DTI) after the onset of consciousness disorders for at least 4 weeks; and (3) patients with CRS-R records within a week before or after the MRI and DTI.

Participants of the stroke without DoC group all met the following criteria: (1) adult patients over 20 years of age who have experienced supratentorial first ever unilateral stroke at least 4 weeks prior; (2) patients with a score of 1 or more on the Korean Mini-Mental State Examination (K-MMSE) after the onset of stroke; and (3) patients who have undergone 3.0-T MRI and DTI at least 4 weeks after stroke onset.

Healthy controls were age and sex matched subjects who had undergone 3.0-T MRI and DTI as part of an advanced health checkup. These individuals did not meet the inclusion criteria for the DoC and stroke without DoC groups and were selected from our institution’s recorded data.

Exclusion criteria for all subjects included: (1) severe brain atrophy or deformation before the onset of the disorder; (2) pre-existing degenerative brain diseases such as dementia or Parkinson’s disease before the onset of the disorder; and (3) life-threatening systemic diseases, severe drug addiction, or alcohol use disorders before the onset of the disorder. The protocols were approved by the Institutional Review Board of our institute, and due to the nature of the study design, the board waived the requirement for informed consent. A flow chart summarizing the inclusion and exclusion criteria for the DoC group has been depicted in Supplementary Figure 1 for clarity.

### Diffusion tensor imaging acquisition

In the healthy control group (*n* = 20), stroke without DoC (*n* = 20) group, and 12 participants from the DoC group, DTI was performed using a 3.0T magnetic resonance imager (MAGNETOM^®^ Verio; Siemens, Erlangen, Germany) equipped with a six-channel head coil. Data were received in the form of single-shot spin-echo echo-planar images, with axial slices covering the whole brain across 76 interleaved slices 2.0 mm in thickness (no gap; repetition time/echo time = 14,300/84 ms; field of view = 224 × 224 mm^2^; matrix 224 × 224; voxel size 1 × 1 × 2 mm^3^ (isotropic); number of excitations = 1). Diffusion sensitizing gradients were used in 64 noncollinear directions with a *b*-value of 1,000 ms/mm^2^. The *b* = 0 images were scanned before obtaining the diffusion-weighted images, with 65 volumes in total. In the remaining 8 participants from the DoC group, DTI was performed with a 3.0T magnetic resonance imager (Phillips Health care, Best, Netherlands) equipped with a 32-channel head coil. Data were received in the form of single-shot spin-echo echo-planar images, with axial slices covering the whole brain across 75 interleaved slices 2.0 mm in thickness [no gap; repetition time/echo time = 1000/75 ms; field of view = 230.4 × 230.4 mm ^2^; matrix = 144 × 144; voxel size = 1.6 × 1.6 × 2 mm^3^ (isotropic), number of excitations = 1]. Diffusion sensitizing gradients were applied in 32 noncollinear directions with a b-value of 1,000 ms/mm^2^. The *b* = 0 images were scanned before acquisition of the diffusion-weighted images, with 33 volumes in total (20–22).

### Image processing

The images were processed using the FMRIB Software Library (FSL; ver. 6.0.5).^1^ Source data were corrected for eddy currents and head motion by registration to the first *b* = 0 image using affine transformation. Probability distributions were then modeled in two fiber directions at each voxel for probabilistic tractography (23). For probabilistic tractography, the BEDPOSTX program, which utilizes a multifiber diffusion model (24), was employed to model probability distributions in two fiber directions at each voxel. Fractional Anisotropy (FA) maps were prepared using the DTIFIT program which fits a diffusion tensor model at each voxel.

### Diffusion tensor tractography

Probabilistic tractography was performed using the FSL Probtrack X program to reconstruct thalamo-dorsolateral prefrontal cortex tract (TDLPFCT). For a precise intragroup comparison within the DoC group, we adjusted the curvature thresholds for correlation analysis. The tracking parameters included: numbers of samples, 5,000; a curvature threshold of 0.5 (cosine 60°, for intergroup analysis among 3 groups) and 0.2 (cosine 78.5°, for intragroup analysis in the DoC group); and a step length of 0.5 mm.

**Figure 1:**
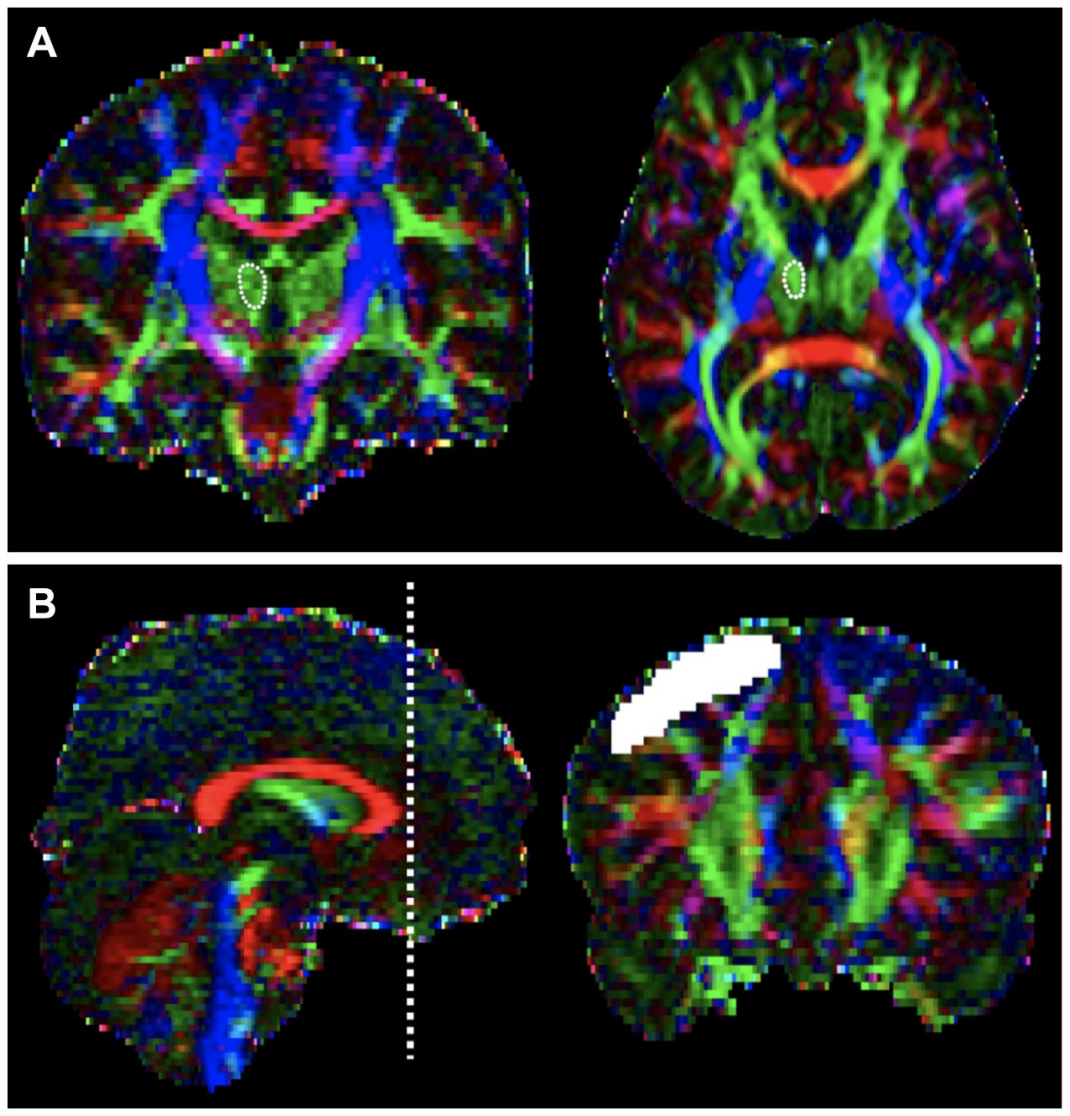
Regions of interest (ROI) for the Thalamo-Dorsolateral prefrontal cortex (DLPFC) tract on color fractional anisotropy (FA) maps of diffusion tensor images. **(A)** Mediodorsal (MD) thalamus as seeds ROI, defined based on the known anatomical location of the thalamus on the axial image. **(B)** The target ROI, DLPFC were drawn on the coronal image. The slice located immediately anterior to the most rostral part of corpus callosum was the first slice of DLPFC ROI, which is depicted on left side. Then tracing from posterior to anterior until the most anterior slice, where the superior and middle frontal gyrus could be distinguished.

Regions of interest (ROIs) were manually defined on a voxel-by-voxel basis for each patient (Figure 1). ROI locations were determined using anatomical landmarks from previous studies and an atlas (8, 25–29). We used a two-ROI approach to reconstruct our tract. The mediodorsal (MD) thalamus was used as the seed ROI, and the DLPFC of the same hemisphere was used as the target ROI. The seed ROI was defined based on the known anatomical location of the thalamus on the axial image (27, 28). The target ROI, encompassed Brodmann areas (BAs) 9 and 46, along with a few transitional areas (29–31), and was drawn on the coronal image. According to previous studies, BA 9 is located in the superior frontal gyrus, and BA 46 is found in the middle frontal gyrus (31). The slice immediately anterior to the most rostral part of the corpus callosum was the starting point for the DLPFC ROI (8). Tracing proceeded from posterior to anterior until reaching the most anterior slice, where the superior and middle frontal gyri could be distinguished (29). On these slices, thick lines were drawn along the cortex from the tip of the superior frontal gyrus to the inferior frontal sulcus to delineate the DLPFC from the rest of the prefrontal cortex.

Tract-based fractional anisotropy (FA) and tract volume (TV) values were calculated from TDLPFCT of each hemisphere using the FSLUTILS program. FA quantifies the degree of directionality of water diffusion in tissues, serving as an indicator of white matter tract integrity. Generally, higher FA values are associated with well-structured and intact white matter, whereas lower FA values may indicate potential damage or degeneration within these neural pathways (32). The FA value of TDLPFCT was calculated by averaging the values of all traced voxels during tracking. The TV value of tract, which represents the total volume of the reconstructed tract used to assess the structural integrity of neural pathways, was calculated by multiplying the voxel volume by the number of traced voxels during tracking, based on a robust minimum intensity threshold of 0.

In the stroke without DoC and DoC groups, the less affected hemisphere was defined as the cerebral hemisphere without a brain lesion, referred to as the unaffected or less affected lobe. However, for some patients in the DoC group with hypoxic injury as the etiology, it was difficult to define the hemisphere with a lesion. Therefore, in these cases and in the healthy control group, the hemisphere showing higher TV values of TDLPFCT compared to the opposite hemisphere was defined as the unaffected or less affected lobe.

### Statistical analysis

To determine whether the DTI-parameters of three groups were normally distributed, the Kolmogorov–Smirnov test and Shapiro–Wilk test were performed. Since some parameters were not normally distributed, the Kruskal-Wallis test was conducted for evaluating the differences among the three groups. This non-parametric test is suitable for comparing three or more independent groups and does not assume normality in the data distribution. For *post hoc* analysis, we conducted the Mann–Whitney *U* test to identify pairwise differences between the groups. To account for multiple comparisons and reduce the risk of Type I errors, we applied the Bonferroni correction. These tests were two-tailed, and a *p*-value of ≤0.0166 was considered statistically significant. Spearman’s rank correlation coefficient was employed to assess the relationship between the TV of the unaffected TDLPFCT and CRS-R. This non-parametric method is appropriate for determining the strength and direction of the association between two variables. A *p*-value of less than 0.05 was considered as statistically significant, indicating a meaningful correlation between the TV of the unaffected TDLPFCT and the level of consciousness as measured by CRS-R scores. All statistical analyses were performed using SPSS software for Windows (ver. 21.0; SPSS, Inc., Chicago, IL, USA).

### Clinical use with a case: the transcranial direct current stimulation for the TDLPFCT

We present a case of a 42-year-old man who received a transcranial direct current stimulation (tDCS) intervention. He was transferred to the Department of Rehabilitation medicine at St. Vincent’s Hospital (Suwon, South Korea) due to a consciousness disorder resulting from hypoxic brain injury following cardiac arrest. The patient underwent a tDCS intervention using a battery-operated, portable device with simulation software (Neurophet innk and tES Lab, Neurophet, Seoul, Republic of Korea) with tES designed to deliver active tDCS. The device administers 2.0 mA of the direct current (with 30 s for ramping up and ramping down) through the two sponge-coated electrodes (electrode size: 5 × 5 cm^2^) (33). The tDCS treatment was performed 10 times over a total of 2 weeks (34).

## Results

### Demographic and clinical characters

Demographic and clinical characteristics of 60 participants from three groups are presented in Table 1. There were no significant differences in age and sex among the three groups. In terms of etiology, the stroke without DoC group consisted of 8 (40%) hemorrhage patients and 12 (60%) cerebral infarction patients. The DoC group included 12 (60%) hemorrhage patients, 3 (15%) infarction patients, and 5 (25%) hypoxic brain injury patients. There were no statistically significant differences in the distribution of more affected side (right or left) between the stroke without DoC group and the DoC group. The median (interquartile range) time of DTI acquisition after onset was 58 (35–352) days for the stroke without DoC group and 61.5 (50–359) days for the DoC group. The mean (±SD) value of CRS-R score (assessed within a week before or after the brain imaging) of the DoC group was 7.4 ± 2.7. More detailed clinical characteristics of the DoC group participants are depicted in Supplementary Table 1.

**Table 1:** Participant’s demographic data.

	Healthy control (*n* = 20)	Stroke without DoC (*n* = 20)	DoC (*n* = 20)	*p*
Age, years	59.6 ± 5.6	58.8 ± 12.8	59.2 ± 12.6	0.067
Female sex, *n* (%)	13 (65%)	12 (60%)	13 (65%)	0.931
Etiology, *n* (%)				
Hemorrhage	–	8 (40%)	12 (60%)	0.206
Infarction	–	12 (60%)	3 (15%)	
Hypoxic brain injury	–	–	5 (25%)	
More affected side, Rt (%)	–	9 (45%)	7 (35%)	0.519
DTI acquisition time after onset (day)	–	58 (35–352)	91.5 (50–359)	0.369
CRS-R score	–	–	7.4 ± 2.7	

Values are means ± standard deviation, median (interquartile range: first-third quartiles), or number (*n*) (%).

*p* values were tested using one-way ANOVA test or Pearson’s chi-square test or Kruskal-Wallis test.

DoC, disorders of consciousness; Rt, Right; DTI, Diffusion tensor imaging; CRS-R, Coma Recovery Scale-Revised.

### Comparison of the DTI-derived parameters among the three groups

Table 2 summarizes the tract volume (TV), fractional anisotropy (FA) of the thalamo-dorsolateral prefrontal cortex tract (TDLPFCT) in each hemisphere, and the fractional anisotropy ratio (rFA) of each group. The TV of the DLPFCT was highest in both the affected and less affected lobes of healthy participants, while the DoC participants had the lowest values in both lobes.

**Table 2:** Values and comparison of TV, FA, rFA of TDLPFCT by group.

		Healthy control (*n* = 20)	Stroke without DoC (*n* = 20)	DoC (*n* = 20)	*p*
TV, mm^3^	Affected	7,616	4,058	187	<0.001*
		7,128 (5,525–9,963)	2,808 (204–7,468)	0 (0–0)	
	Less affected	11,716	7,053	1,529	<0.001*
		12,424 (9,533–13,316)	8,084 (2,504–9,613)	97 (0–2,440)	
FA	Affected	0.35	0.30	0.11	0.002*
		0.35 (0.33–0.37)	0.33 (0.31–0.34)	0.00 (0.00–0.10)	
	Less affected	0.33	0.35	0.21	0.074
		0.33 (0.31–0.35)	0.35 (0.33–0.37)	0.28 (0–0.37)	
rFA (affected/less affected)		1.06 (1.02–1.11)	0.85	0.17	<0.001*
			0.93 (0.87–0.99)	0.00 (0.00–0.00)	

Values are represented as both means and median (interquartile range: first-third quartiles). *p < 0.05, three groups were compared using Kruskal-Wallis test.

TV, Tract volume; FA, Fractional anisotropy; rFA, fractional anisotropy ratio (affected/less affected); TDLPFCT, Thalamo-Dorsolateral prefrontal cortex tract; DoC, Disorders of Consciousness.

**Figure 2:**
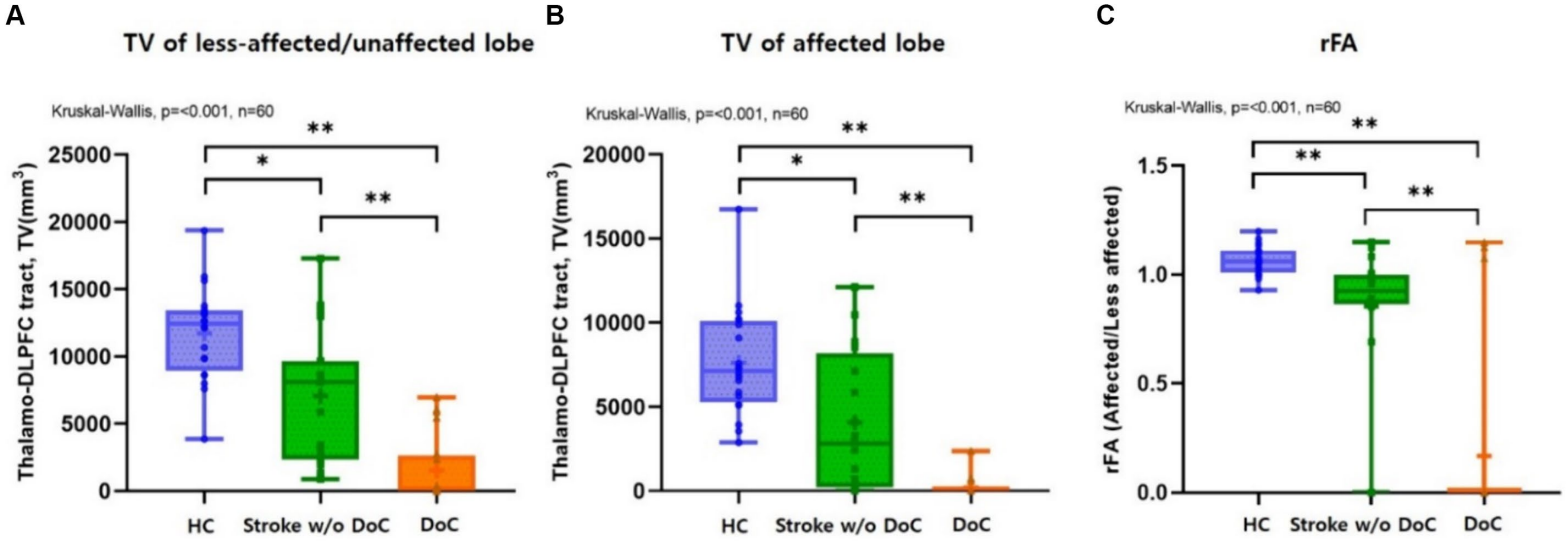
Box plots comparing the **(A)** TDLPFC tract volume on less-affected/or unaffected and **(B)** affected sides showed significant differences (*p* < 0.001) among the three groups, respectively, with the greatest reduction observed in DoC participants. Box plots comparing the **(C)** rFA (fractional anisotropy ratio) of TDLPFC tract among three groups, which showed significant differences (*p* < 0.001). *Post hoc* analysis confirmed significant intergroup differences. DLPFC, Dorsolateral prefrontal cortex; TV, Tract volume; rFA, Fractional anisotropy ratio; HC, healthy control; w/o, without; TDLPFC, Thalamo-dorsolateral prefrontal cortex; *p value <0.01 **p value <0.001.

The TV of the DLPFCT differed significantly among the three groups in both the affected and less affected lobes (*p* < 0.001). Subgroup analysis using Mann–Whitney *U*-test with Bonferroni correction (*p* ≤ 0.0166 deemed significant) was performed, and box plots comparing the TDLPFCT volume were depicted in Figure 2. All pairwise comparisons between the groups (Healthy & DoC, Healthy & Stroke without DoC, Stroke & DoC) showed statistical differences, with the DoC group exhibiting the greatest reduction in both the affected and less affected hemispheres.

**Figure 3:**
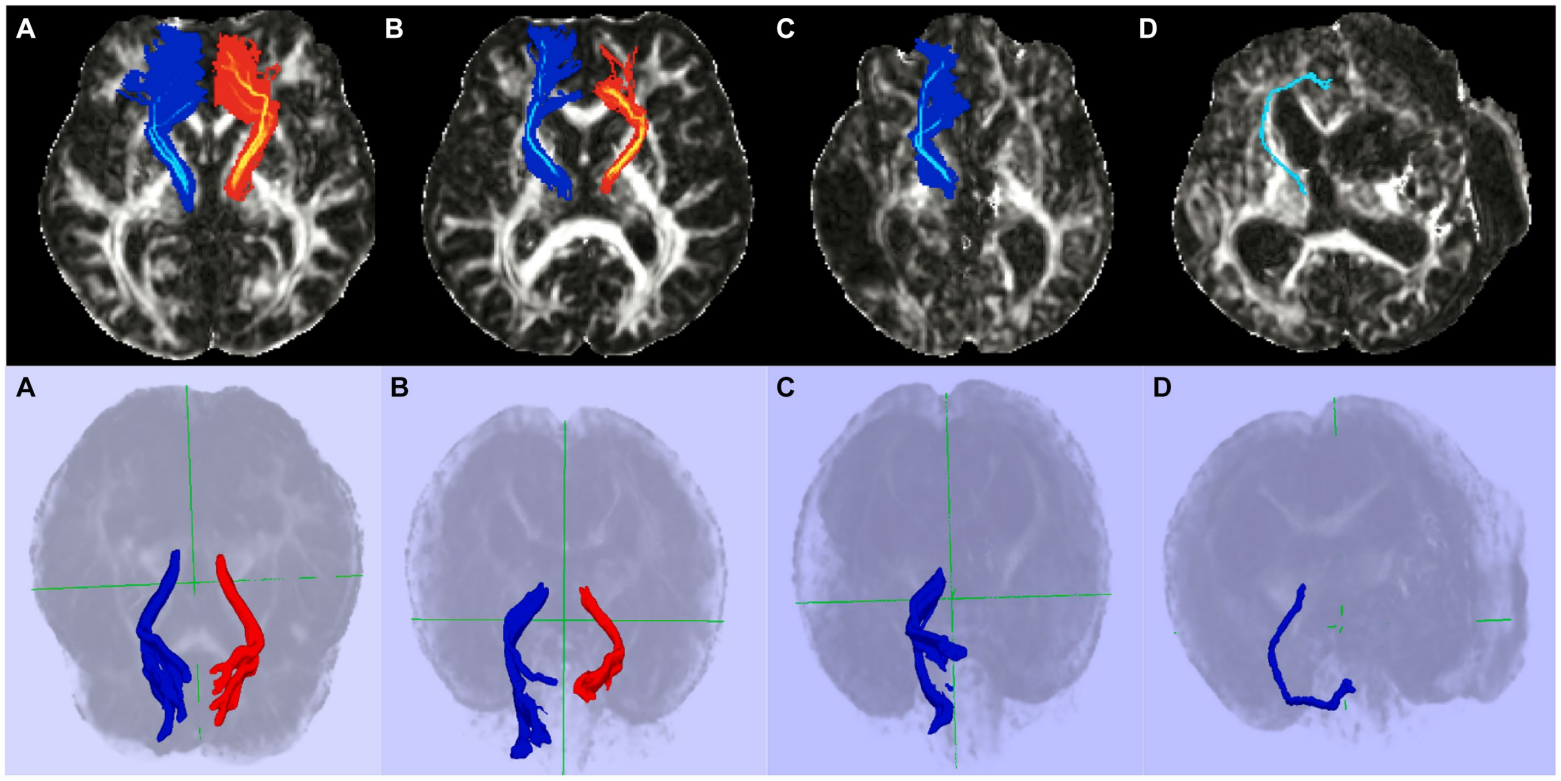
Representative diffusion tensor tractography images of the TDLPFCT. **(A)** Healthy control, **(B)** Stroke without disorders of consciousness, **(C)** Disorders of consciousness with relatively high CRS-R, **(D)** Disorders of consciousness with relatively low CRS-R. Additional coronal, and sagittal images are depicted on Supplementary material. TDLPFCT, Thalamo-dorsolateral prefrontal cortex tract; CRS-R, Coma recovery scale- revised.

The FA value was highest in both lobes for the healthy controls and lowest in the DoC group. There was a significant difference in the FA value of the affected TDLPFCT among the three groups (*p* = 0.002). However, there was no significant difference among the three groups in the less affected lobe for the FA value of the TDLPFCT (*p* = 0.074). Additionally, we calculated the FA ratio (rFA), which represents the ratio of the FA value of the TDLPFCT in the affected side to the FA value of the less affected lobe. The mean and median values of rFA were highest in the healthy control group and lowest in the DoC group, showing a significant difference (*p* < 0.001). *Post hoc* analysis revealed significant differences in all combinations of the two groups, as shown in the box plot in Figure 2. Representative DTI images of the TDLPFCT are depicted in Figure 3.

### Correlation between TV and residual consciousness in DoC group

**Figure 4:**
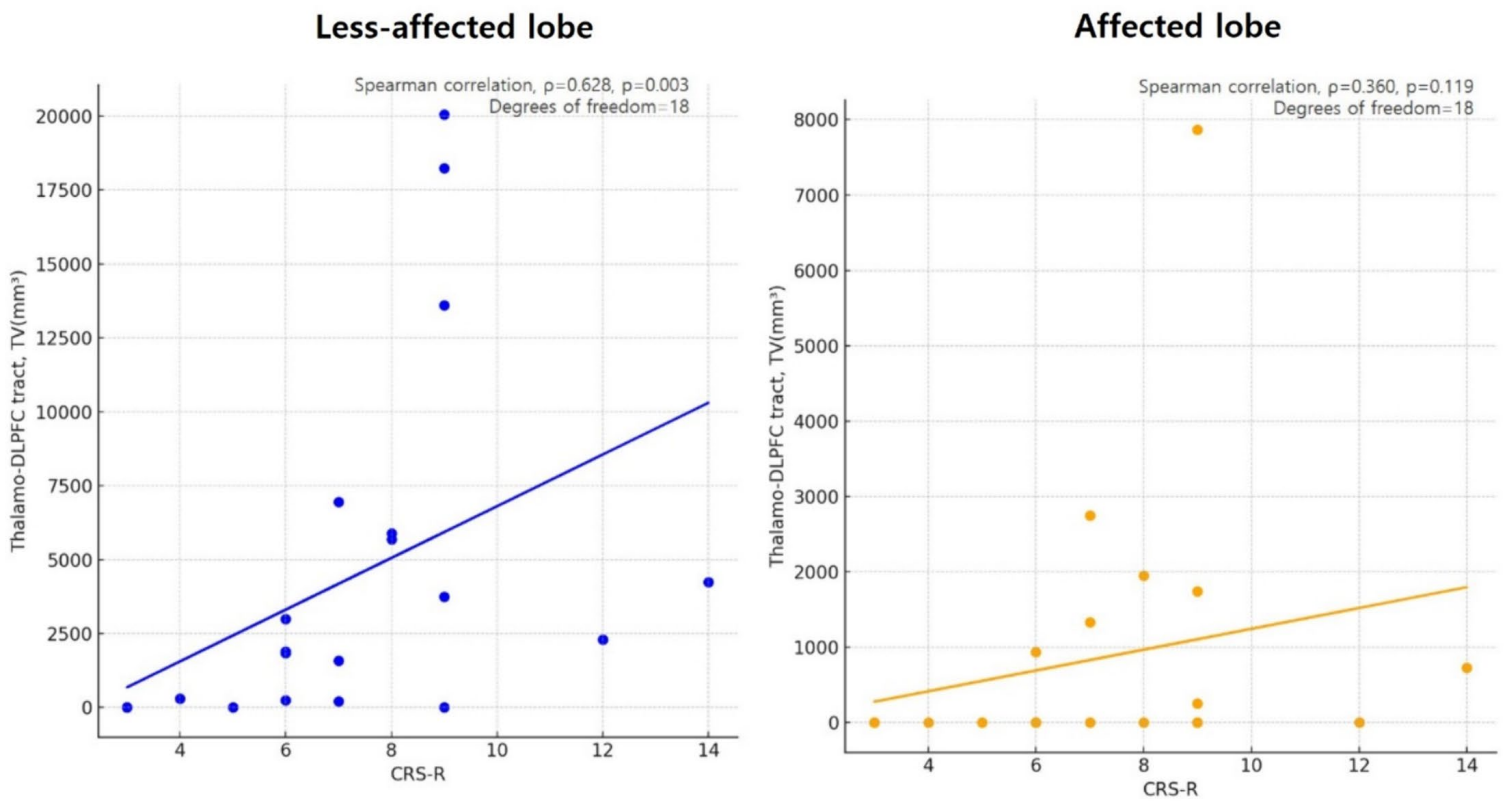
Scatter plot showing the correlation of less affected/or unaffected Thalamo-DLPFC tract volume and CRS-R in disorders of consciousness (DoC) participants, using Spearman’s correlation coefficient (*ρ* = 0.628, *p* = 0.003, degrees of freedom = 18). However there were no significant correlation between Thalamo-DLPFC tract volume of affected lobe in DoC participants and CRS-R (*ρ* = 0.360, *p* = 0.119, degrees of freedom = 18). DLPFC, Dorsolateral prefrontal cortex; TV, tract volume; CRS-R, Coma recovery scale-revised.

A significant correlation was found between the TV of the TDLPFCT in the unaffected lobe, indicating more preserved TDLPFCT, and the CRS-R score, as indicated by a Spearman’s correlation coefficient of *ρ* = 0.628 (*p* = 0.003, degrees of freedom = 18). However, there was no significant correlation between the TV of the affected lobe and the CRS-R score, with a correlation coefficient of *ρ* = 0.360 (*p* = 0.119, degrees of freedom = 18). The scatter plot illustrating this correlation is shown in Figure 4.

### A case with transcranial direct current stimulation for residual TDLPFCT

**Figure 5:**
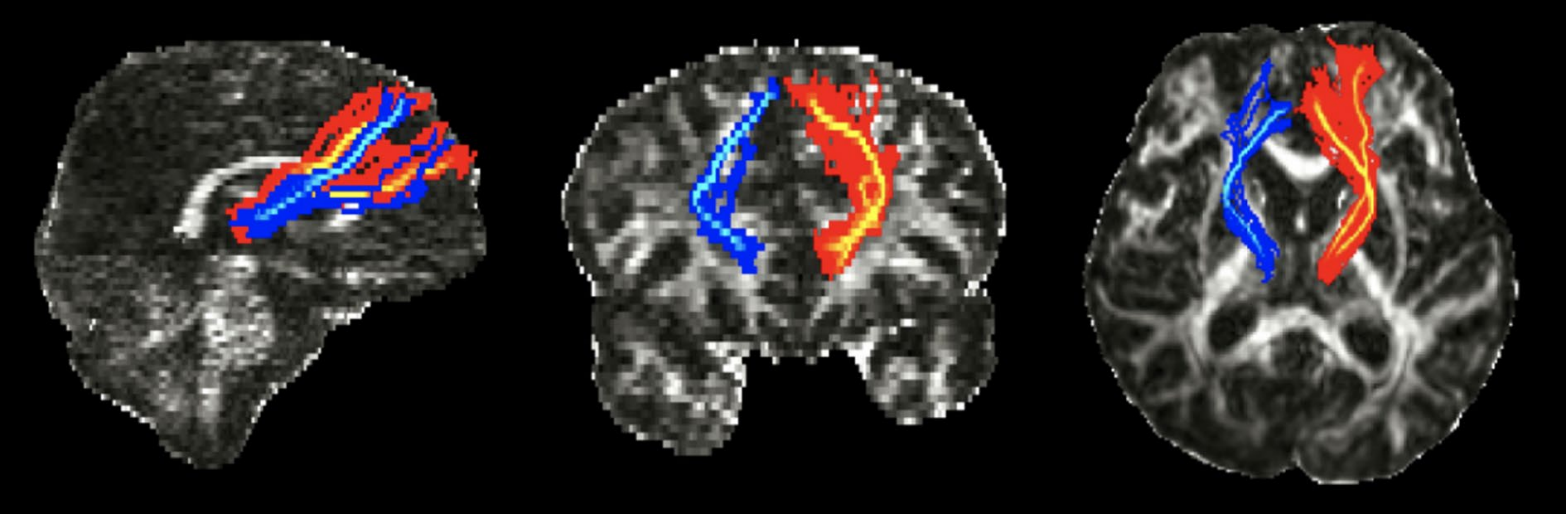
Diffusion tensor tractography images of the TDLPFCT of 42-year-old patient with consciousness disorder. TDLPFCT, Thalamo-dorsolateral prefrontal cortex tract.

To support our hypothesis regarding the significance of the integrity of the TDLPFCT in patients with DoC, we present a compelling case of a 42-year-old man who demonstrated significant improvements following transcranial direct current stimulation (tDCS) targeted at the relatively preserved left DLPFC. He was diagnosed with MCS, and his initial CRS-R score was 5 (A1V0M2O0C0Ar2). We assessed the integrity of the DLPFCT and found that the left DLPFCT was relatively preserved DLPFC (Figure 5). Based on the patient’s MRI, we applied the transcranial direct current to target the less-affected, left DLPFC via Neurophet’s tES Lab (35, 36). Following 10 consecutive tDCS sessions, the patient woke up with a CRS-R score of 18 (A4V4M4O2C1Ar3). Two months later, he began demonstrating partial oral eating and was able to sit with partial assistance.

## Discussion

Our hypothesis revolves around the idea that the remaining integrity of the thalamo-dorsolateral prefrontal cortex tract (TDLPFCT) may play a pivotal role in determining consciousness levels among patients with prolonged Disorders of Consciousness (DoC). Consequently, our findings proved a positive correlation between the remaining integrity of TDLPFCT, specifically the tract volume (TV) of the less affected TDLPFCT, and the level of consciousness in DoC patients. Additionally, our results revealed a stepwise decrease in the remaining integrity of TDLPFCT among the three groups: healthy control, stroke without DoC, and DoC group. These observations suggest that TDLPFCT may be a critical anatomical structure influencing the level of consciousness in DoC patients and remaining integrity of TDLPFCT can potentially serve as a valuable biomarker for assessing their consciousness state.

The development of probabilistic tractography, using diffusion tensor imaging (DTI), has facilitated the estimation, visualization, and quantitative analysis of specific neural tracts (24, 37, 38). Several studies have used DTI to investigate white matter abnormalities in DoC patients, showing impairments in the white matter tracts connecting the striatum, thalamus, frontal cortex, and other cerebral cortex areas (39–43). Another DTI study demonstrated a reduction in white matter fibers of the thalamus in DoC patients compared to controls (44), while a subsequent study revealed significant differences in thalamic projections to cerebral cortices, including the frontal cortex, between chronic DoC patients and those who have recovered from DoC (45). Based on evidence from previous studies, it is clear that evaluating the integrity of the connectivity between the MD thalamus and the DLPFC offers a promising approach to assess residual consciousness in patients with prolonged DoC, as demonstrated by the results of our study.

The present study provides novel insights into thalamo-cortical connectivity and its relation to consciousness in patients with prolonged DoC. We compared thalamo-cortical connectivity among three groups, namely healthy controls, stroke patients without DoC, and DoC patients. Our findings demonstrate significant changes in connectivity between the thalamus and the dorsolateral prefrontal cortex (DLPFC) in DoC patients, indicating the potential involvement of the thalamo-cortical pathway in the mechanisms underlying consciousness.

To our knowledge, no prior studies have directly compared thalamo-cortical connectivity among these three groups. We observed a substantial reduction in the connectivity of the affected and less affected thalamo-DLPFC tracts in the DoC group compared to the other groups. Since there were only 2 (10%) participants of stroke without DoC group had 0 value of DTI parameter (such as FA, TV) in affected lobe, 17 (85%) participants of DoC group showed 0 value of TV and FA in affected TDLPFCT, which implies TDLPFCT plays significant role in consciousness.

A previous study by Cosgrove et al. (8) found that the integrity of certain thalamo-prefrontal connections is significantly associated with early command-following in TBI patients. However, DLPFC connectivity to the thalamus was not significantly associated with command-following in TBI patients, which is quite different from our findings. This contrast is intriguing and highlights the potential variability in the role of thalamo-DLPFC connectivity across different etiologies of consciousness disorders.

Furthermore, we found a positive correlation between the integrity of the less affected TDLPFC tract, potentially representing residual function, and the level of consciousness in DoC patients. This suggests that assessing the integrity of the less affected TDLPFCT could serve as a potential prognostic tool for evaluating residual consciousness in DoC patients. Reliable prognostication tools for DoC patients are currently lacking, and individualized assessment of patients’ characteristics and potential therapeutic response is essential (1). Therefore, the measuring TDLPFCT integrity in the less affected hemisphere could be considered a novel predictor for the prognosis of DoC, providing valuable information on the residual consciousness level.

Our focus on the less affected thalamo-DLPFC tract is particularly groundbreaking, as it could pave the way for new perspectives in the treatment of DoC patients. Managing patients with DoC presents significant challenges due to their limited communication and interaction with the environment, raising ethical concerns (46, 47). Current therapeutic approaches for DoC patients mainly rely on interventions that do not require active patient participation. Non-invasive brain stimulation techniques, such as transcranial direct current stimulation (tDCS) (14, 48–52) and repetitive transcranial magnetic stimulation (rTMS) (53–56) have emerged as relatively safe treatment options for DoC patients. These techniques offer potential benefits in promoting recovery and enhancing neuroplasticity in individuals with DoC (47). However, the application of non-invasive brain stimulation may encounter difficulties in cases where the affected side is involved, such as patients with craniectomy, cranioplasty using metal implants, or coil embolization. By focusing on reinforcing the integrity of the less affected thalamo-DLPFC tract, it is conceivable that non-invasive brain stimulation techniques could be applied to a larger number of DoC patients. This perspective introduces a new spectrum of treatment possibilities for DoC patients.

Another strength of our study is the inclusion of prolonged DoC patients with diverse etiologies, including hemorrhage, infarction, and hypoxic brain injury. Considering that the mechanisms underlying DoC can vary depending on the etiology, each of which may differentially impact the integrity of the TDLPFCT. Previous studies have suggested that the pathophysiological mechanisms underlying DoC differ based on the cause of injury, which in turn can influence the degree and location of white matter damage (57). For instance, TBI typically results in diffuse axonal injury, which may disrupt multiple neural pathways, whereas stroke often leads to more focal lesions. To better understand these differences, subgroup analyses to explore the impact of different etiologies on TDLPFCT integrity and its correlation with residual consciousness should be conducted. These analyses, however, are limited by the small sample sizes within each etiology subgroup, and future studies should aim to recruit a larger number of participants specific to each etiology to validate the findings of this study.

As we briefly presented a 42-year-old male patient with a consciousness disorder stemming from hypoxic brain injury post-cardiac arrest, we found that targeting the less affected DLPFC in treatment could be promising for patients with DoC. Although this finding is based on a single case, its practical validity will need to be further validated through future randomized controlled trials (RCTs) and clinical investigations. Nevertheless, our study results indicate a potential direction for the treatment of patients with disorders of consciousness, offering hope for advances in this field.

However, there are certain limitations to our study. Firstly, the sample size was relatively small with only 20 participants in each group. To further substantiate our hypothesis, larger studies with increased participant numbers are essential. Additionally, the participants with DoC were recruited from two different institutions, which led to variations in MRI machines and settings. This discrepancy in diffusion image quality settings between the institutions may have introduced confounding factors that could affect the interpretation of the results. Future studies should strive to include a larger number of participants from a single institution to minimize these confounding effects.

In the context of current research, prolonged DoC is considered quite a rare clinical presentation (58, 59). This scarcity often limits studies to smaller scales or case series, mirroring a pragmatic approach to research on this condition. While the inherent limitations in sample sizes may introduce biases, these are often not seen as significant detriments given the infrequency of prolonged DoC. Nonetheless, the prospect of conducting a large-scale cohort studies are necessary to reinforce and validate our findings. Such a comprehensive approach could effectively address and potentially overcome the inherent biases.

The pathophysiology of DoC is complex and involves intricate networks among brain white matter and deep cortical structures. It is well-established that the lack of consciousness observed in disorders of consciousness (DoC) is associated with significant imbalances in the anterior forebrain mesocircuit system. Specifically, these imbalances involve excessive inhibition within the thalamus and insufficient excitation of the cortical regions (6). According to the mesocircuit model proposed by Schiff, this disruption arises due to widespread deafferentation and neuronal loss, which collectively reduce excitatory neurotransmission and overall synaptic activity. Such alterations critically undermine the thalamocortical pathways’ role in maintaining arousal and cognitive functions, thus contributing to the impaired consciousness characteristic of DoC.

However, not only the mesocircuit system but also several brain networks, including the executive control, default mode, dorsal attention, and salience networks, are correlated with the level of consciousness and interact with the mesocircuit system (60, 61). Among these brain networks, the default mode network (DMN), which involves key regions such as the medial prefrontal cortex (mPFC) and posterior cingulate cortex (PCC), is the most studied in prolonged DoC. However, the executive control network (ECN), which includes the DLPFC and posterior parietal cortex (PPC) as key regions, has also been found to be impaired in prolonged DoC (61). As indicated in various previous studies, the DLPFC is a common target for neuromodulation therapy in patients with prolonged DoC, aiming to restore the disrupted balance of activation between the ECN and DMN by enhancing dopaminergic-driven connectivity (62). Our results support the importance of the DLPFC in explaining the mechanisms and prognosis of DoC. Building on previous studies, further research focusing on the connectivity between structures such as the mPFC or PCC and the thalamus could enhance our understanding of DoC.

Identifying which anatomical structures and tracts among the numerous brain structures involved in DoC are crucial is valuable for evaluating patient prognosis and advancing the development of treatment approaches. This study provides evidence supporting the significant role of thalamus-DLPFC connectivity, particularly in the less affected hemisphere, particularly in the less affected hemisphere, highlighting its potential importance in assessing prognosis and further developing therapeutic strategies for patients with DoC.

## Conclusion

The findings suggest that the TDLPFCT plays an important role in maintaining consciousness, and the integrity of the less affected TDLPFCT is positively correlated with the residual consciousness level, making it a potential predictor for the prognosis of DoC patients.

## Data availability statement

The data presented in this study are available on request from the corresponding author in compliance with the requirements of the data review board of the institution and the personal information protection act.

## Ethics statement

The studies involving humans were approved by the Institutional Review Board of Catholic University, College of Medicine. The studies were conducted in accordance with the local legislation and institutional requirements. The participants provided their written informed consent to participate in this study.

## Author contributions

JJ: Conceptualization, Data curation, Investigation, Visualization, Writing – original draft, Writing – review & editing. YY: Formal analysis, Investigation, Methodology, Resources, Writing – original draft. M-JY: Data curation, Resources, Writing – original draft. BH: Supervision, Validation, Writing – original draft. T-WK: Conceptualization, Writing – original draft. G-YP: Supervision, Writing – original draft. JL: Supervision, Writing – original draft. S-HL: Data curation, Writing – original draft. SI: Conceptualization, Resources, Validation, Writing – original draft. SL: Conceptualization, Data curation, Funding acquisition, Investigation, Resources, Supervision, Visualization, Writing – original draft, Writing – review & editing.

## Footnotes

^1^ http://www.fmrib.ox.ac.uk/fsl
