# Supplementary material for: The integrity of thalamo-dorsolateral prefrontal cortex tract: a key factor in residual consciousness in disorders of consciousness patients
Source: Front Neurol. 2024 Aug 14;15:1373750. doi: 10.3389/fneur.2024.1373750 (PMC11349516; doi:10.3389/fneur.2024.1373750)
Supplement: Supplementary file 1 [file Data_Sheet_1.docx]

**Supplementary Fig 1.** Flow chart detailing the inclusion and exclusion criteria for the DoC group in the study. Abbreviations: DoC, Disorders of consciousness; MRI, Magnetic resonance imaging; DTI, Diffusion tensor imaging; CRS-R, Coma recovery scale- revised.


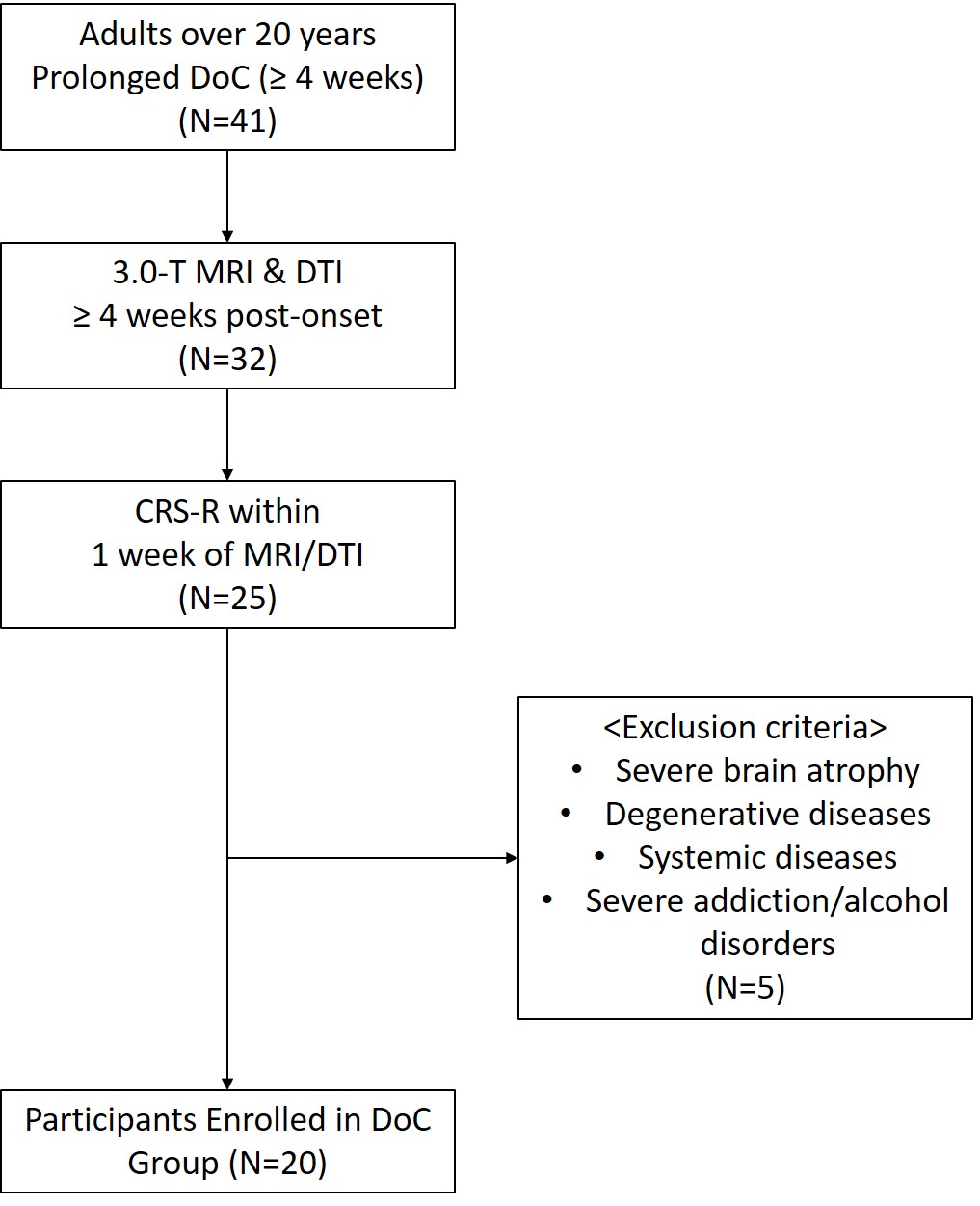


**Supplementary Fig 2.** Representative diffusion tensor tractography images of the TDLPFCT. (A) Normal control, (B) Stroke without disorders of consciousness, (C) Disorders of consciousness with relatively high CRS-R, (D) Disorders of consciousness with relatively low CRS-R. Abbreviations: TDLPFCT, Thalamo-dorsolateral prefrontal cortex tract; CRS-R, Coma recovery scale- revised.


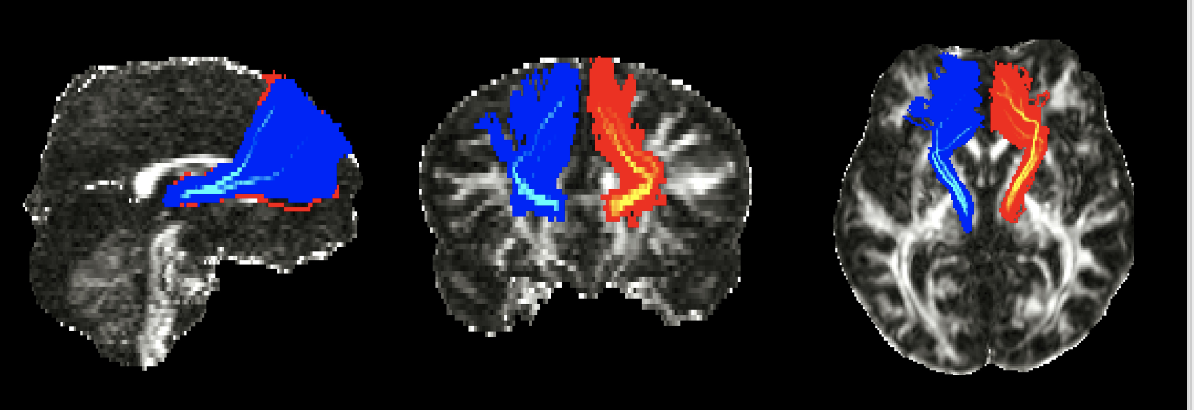

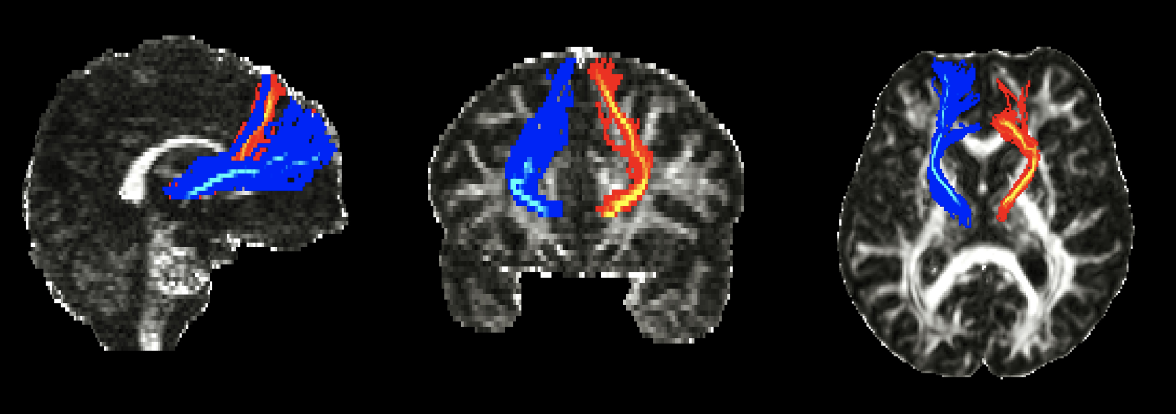

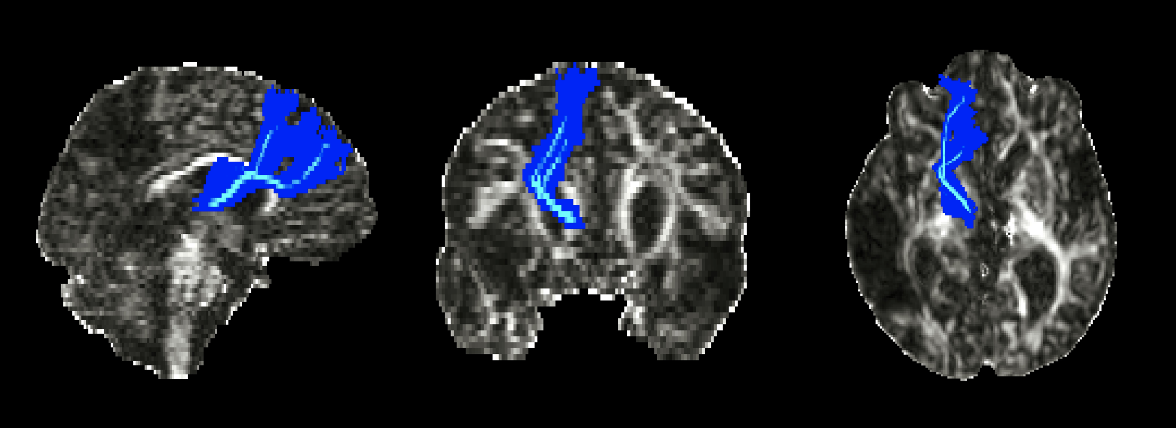

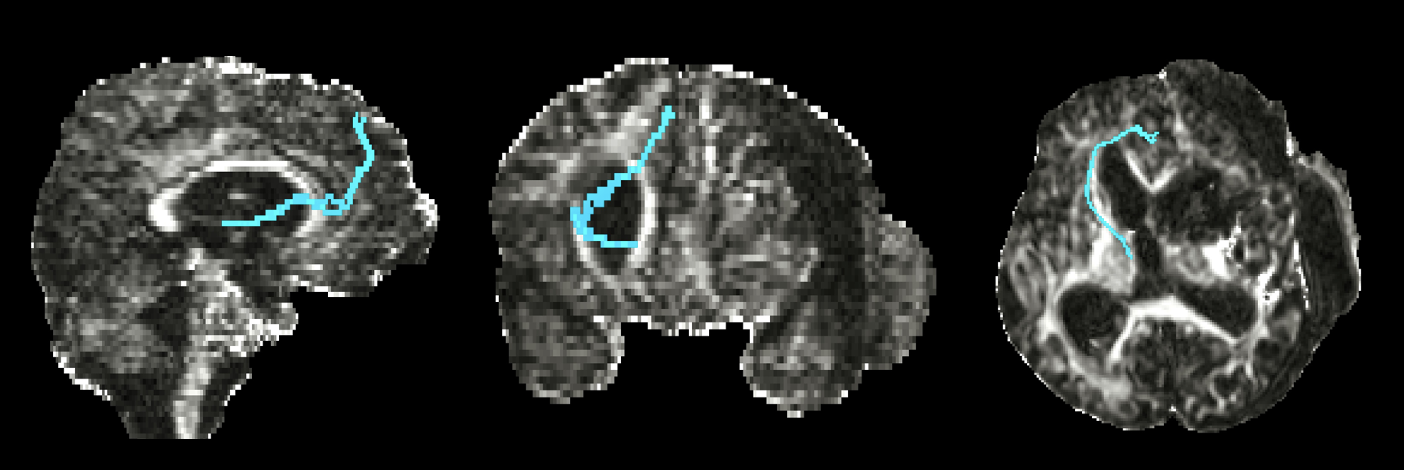


**(A)**

**(B)**

**(C)**

**(D)**

**Supplementary Fig 3.** Representative diffusion tensor tractography 3-dimentional images of the TDLPFCT. (A) Normal control, (B) Stroke without disorders of consciousness, (C) Disorders of consciousness with relatively high CRS-R, (D) Disorders of consciousness with relatively low CRS-R. Abbreviations: TDLPFCT, Thalamo-dorsolateral prefrontal cortex tract; CRS-R, Coma recovery scale- revised.


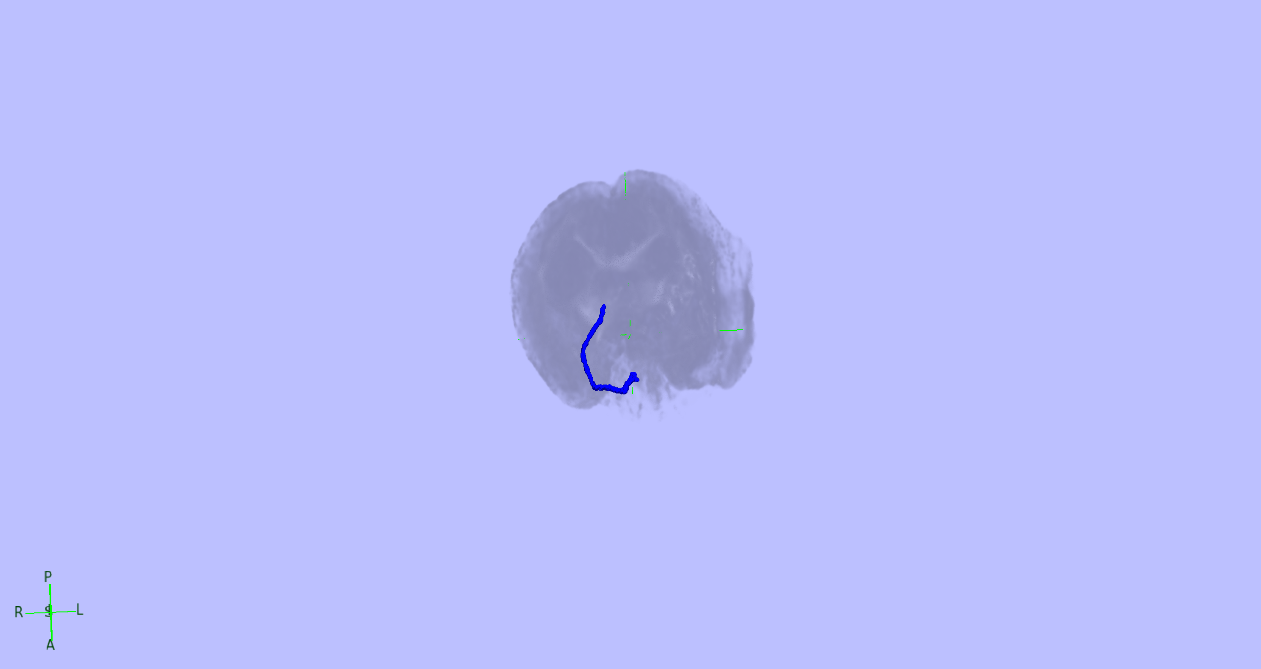

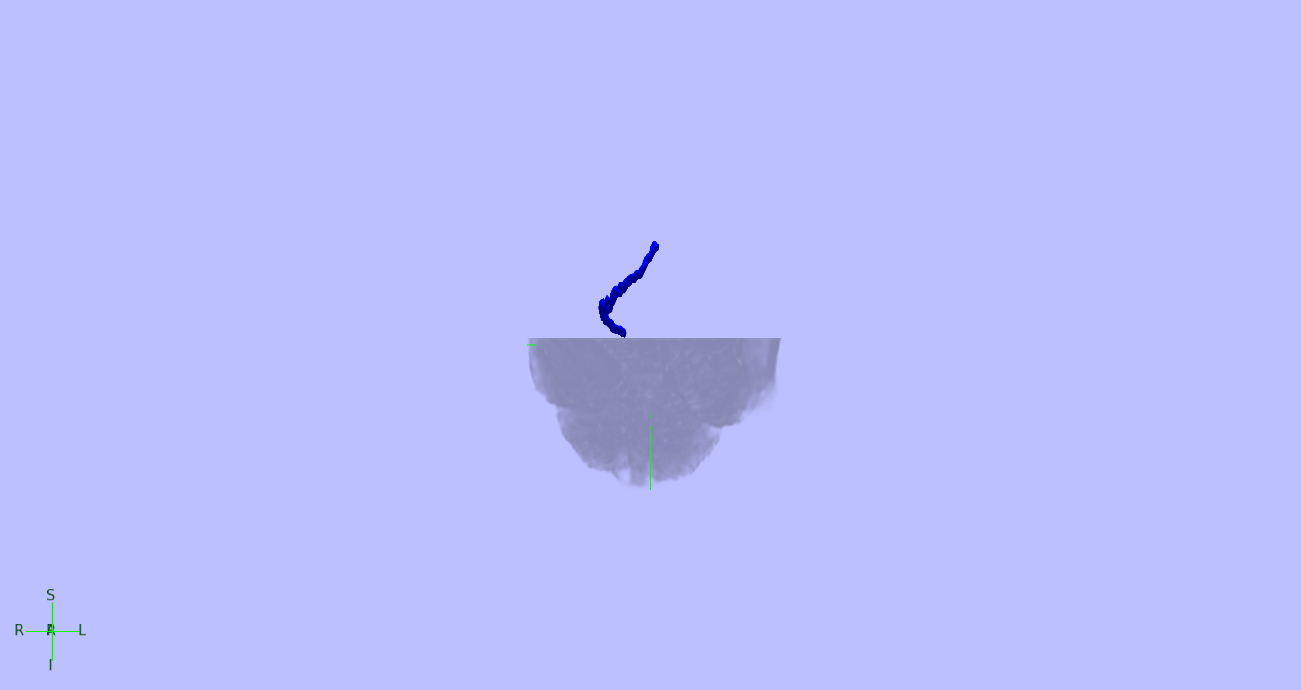

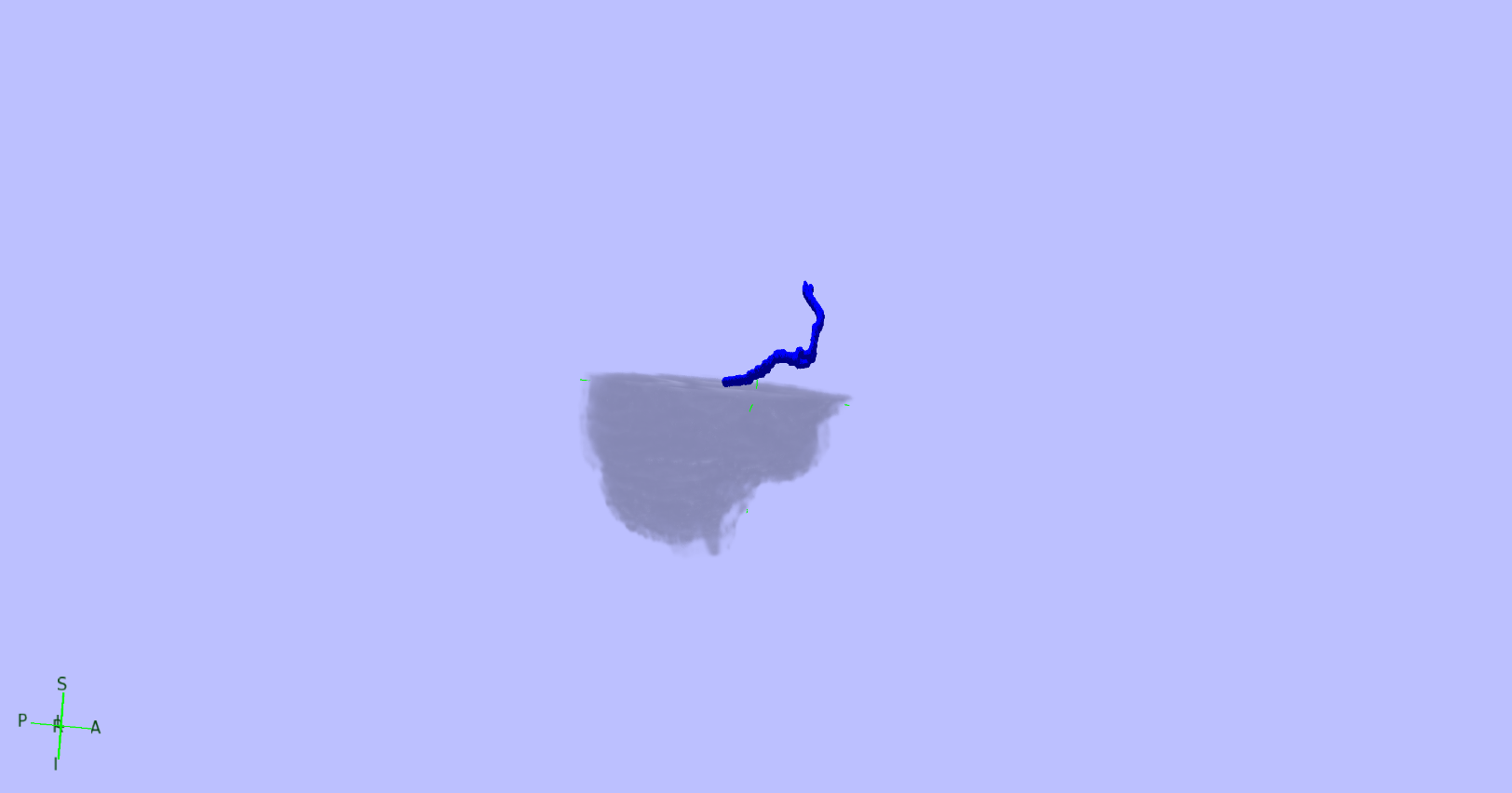

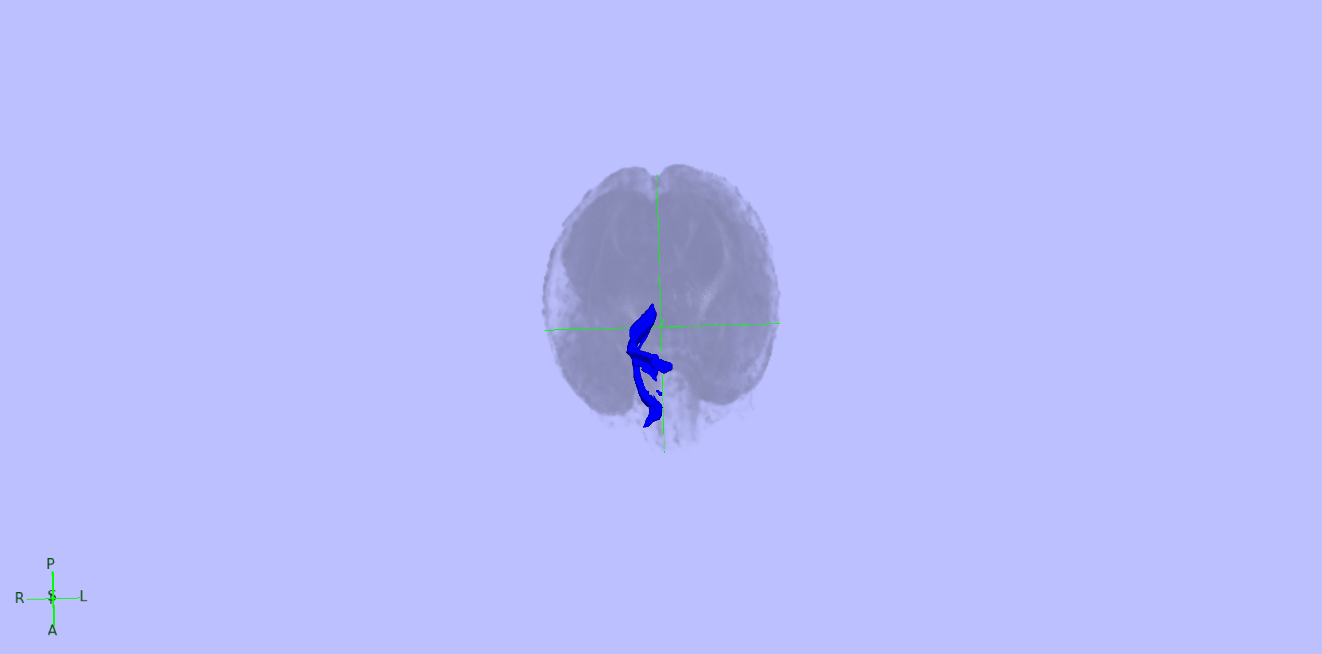

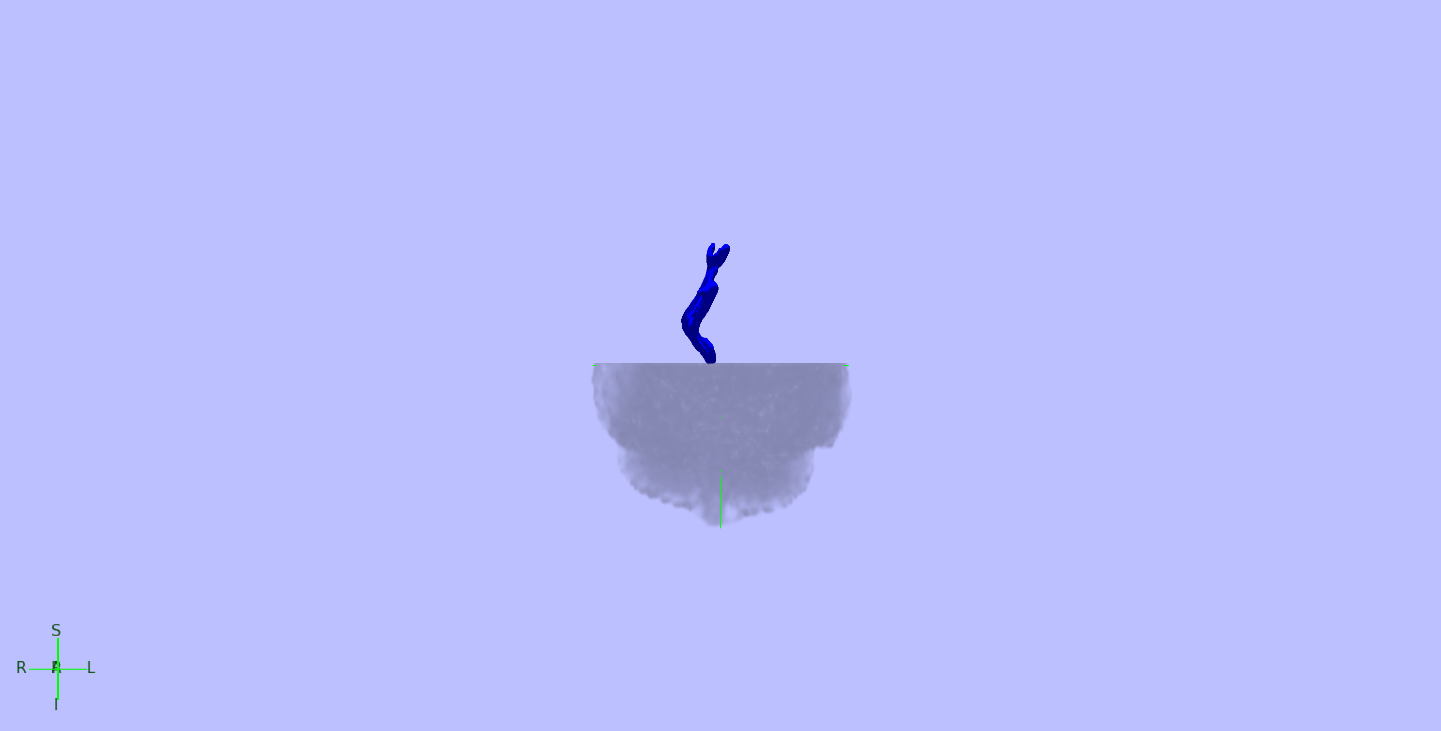

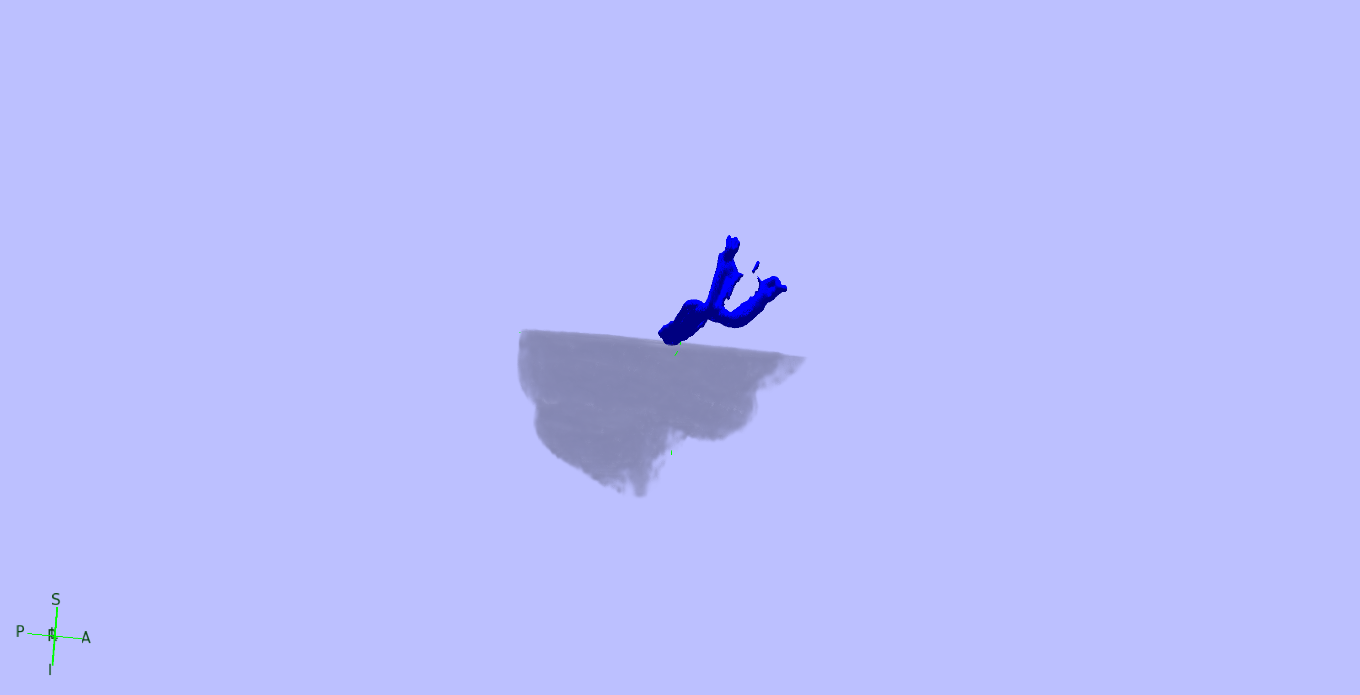

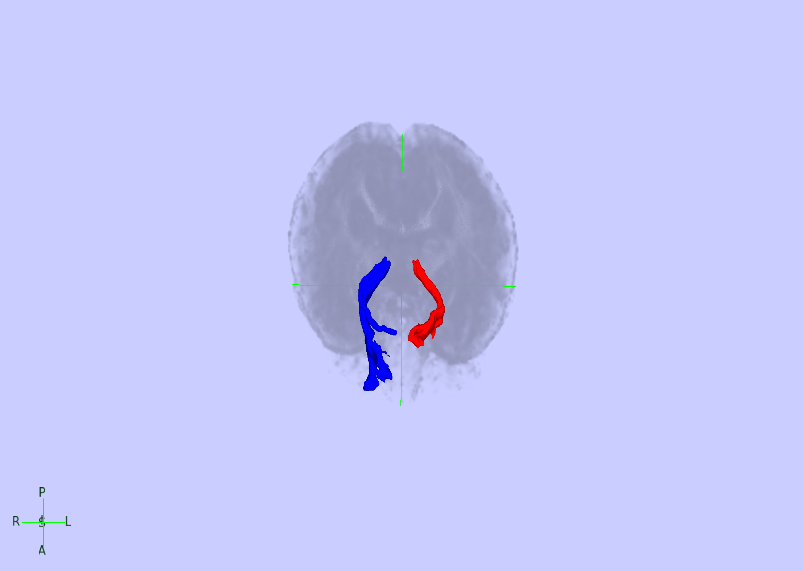

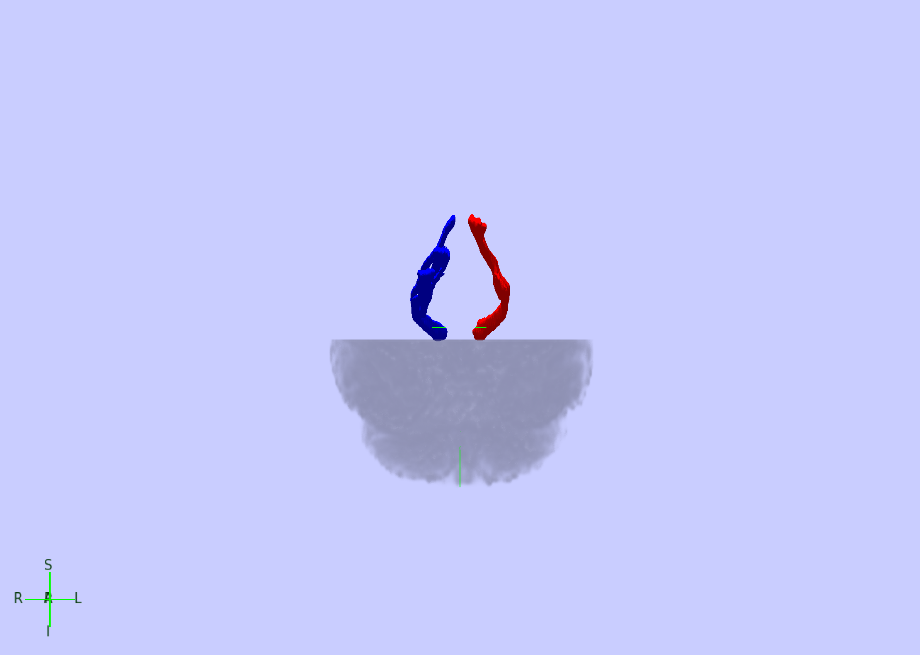

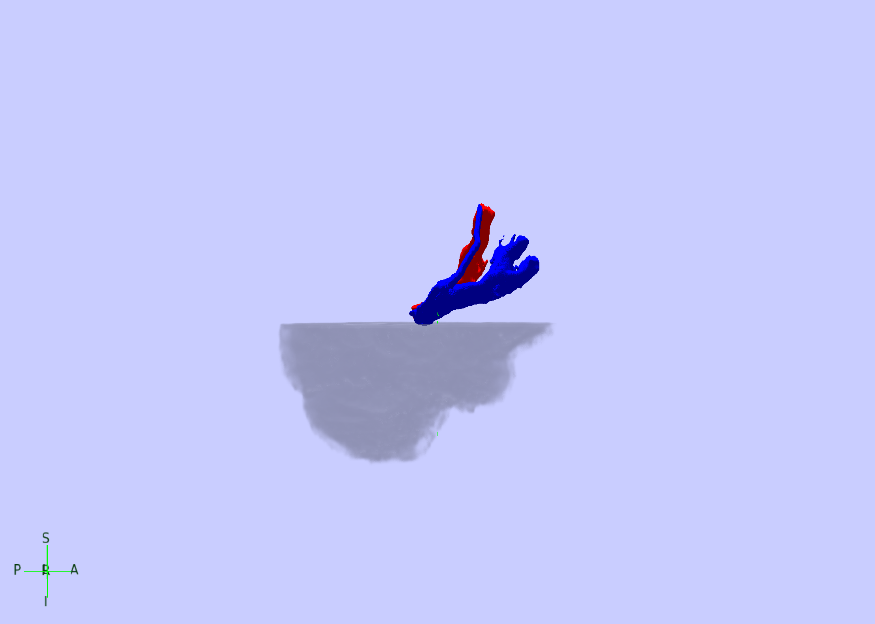

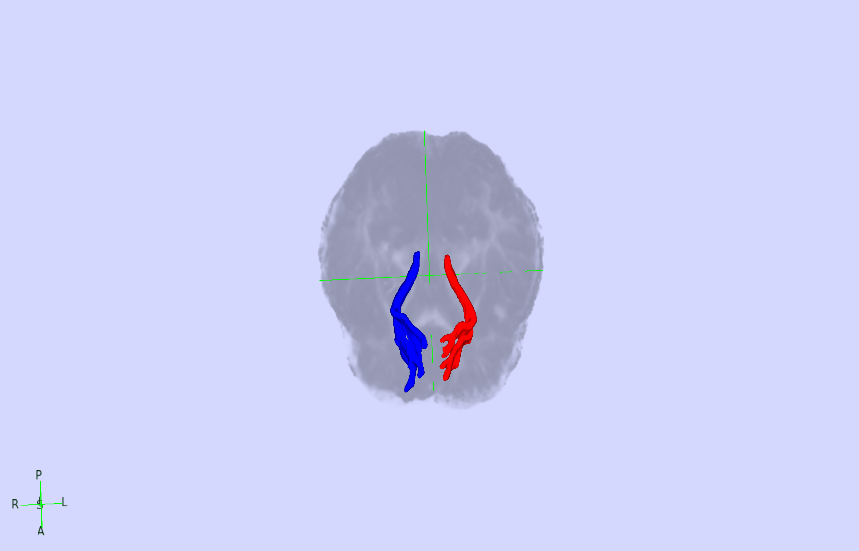

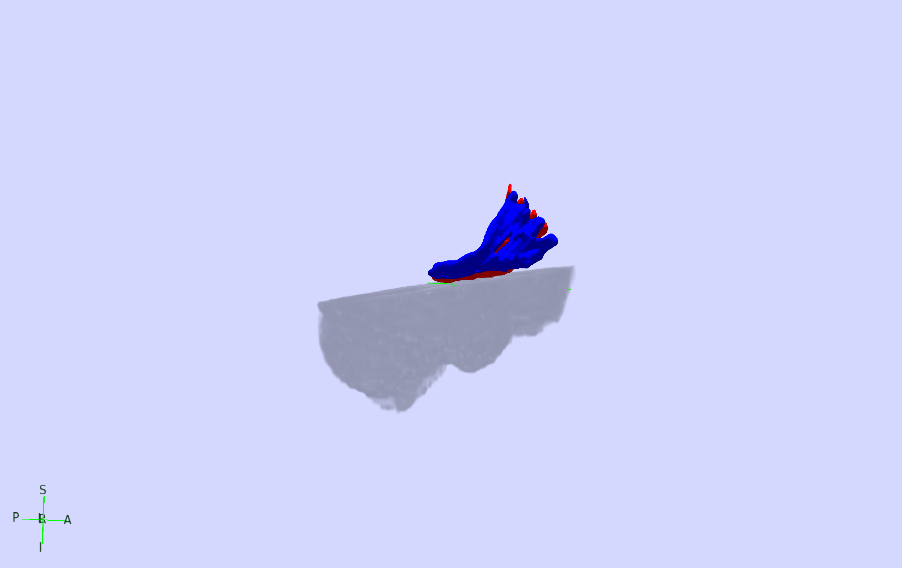

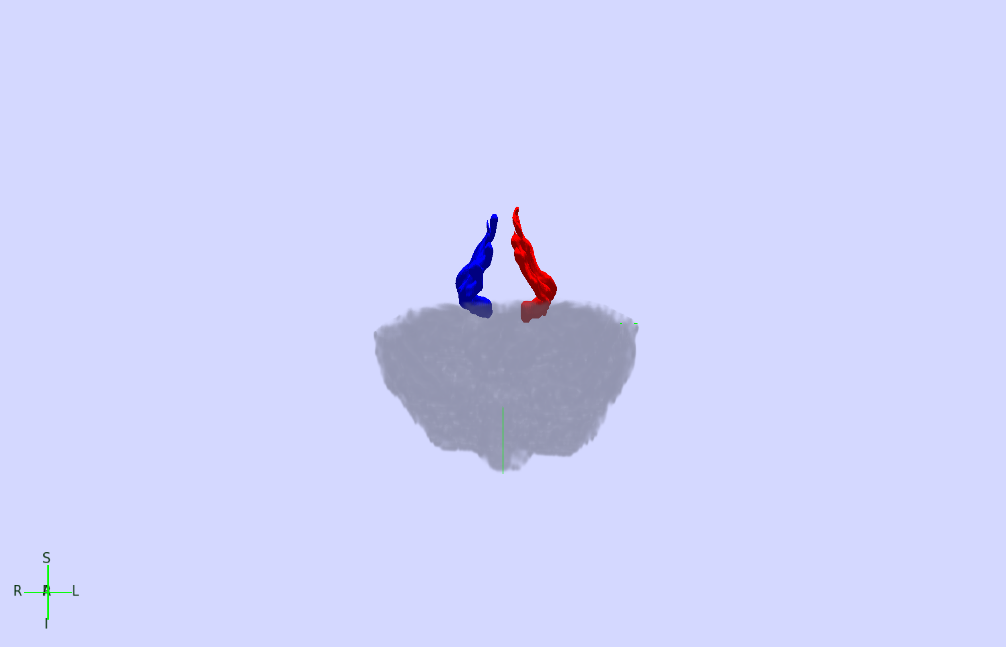


**(A)**

**(B)**

**(C)**

**(D)**

**Supplementary Table 1.** Clinical characteristics of DoC group participants.

| **Subject**  **number** | **Age** | **Sex** | **Epidemiology** | **Imaging findings** | **Location** | **More affected sided** | **CRS-R** |
| --- | --- | --- | --- | --- | --- | --- | --- |
| **1** | 42 | M | Hypoxic | DAI |  | Rt. | 9 |
| **2** | 47 | M | Hemorrhage | ICH | Rt. Frontal, BG | Rt. | 5 |
| **3** | 62 | M | Hemorrhage | ICH, IVH | Lt. thalamus | Lt. | 9 |
| **4** | 64 | M | Hypoxic | DAI |  | Rt. | 6 |
| **5** | 64 | M | Hemorrhage | ICH | Lt. BG | Lt. | 6 |
| **6** | 68 | M | Infarction | Infarction with HT | Lt. PCA infarction | Lt. | 14 |
| **7** | 71 | M | Infarction | Infarction with ICH | Rt. MCA infarction, left frontal ICH | Rt. | 8 |
| **8** | 30 | F | Hypoxic | DAI |  | Rt. | 7 |
| **9** | 46 | F | Hypoxic | DAI |  | Lt. | 3 |
| **10** | 47 | F | Hemorrhage | ICH | Lt. frontal | Lt. | 6 |
| **11** | 53 | F | Hemorrhage | ICH | Lt. thalamus | Lt. | 9 |
| **12** | 54 | F | Hypoxic | DAI |  | Rt. | 3 |
| **13** | 55 | F | Hemorrhage | ICH, IVH | Lt. BG | Lt. | 9 |
| **14** | 57 | F | Hemorrhage | ICH, IVH | Lt. parieto-occipital | Lt. | 6 |
| **15** | 77 | F | Hemorrhage | ICH, IVH | Lt. thalamus | Lt. | 7 |
| **16** | 64 | F | Hemorrhage | SAH | Lt. DACA ruptured aneurysm | Lt. | 9 |
| **17** | 62 | F | Hemorrhage | SAH | Lt. Pcom ruptured aneurysm | Rt. | 7 |
| **18** | 66 | F | Hemorrhage | ICH | Lt. BG | Lt. | 12 |
| **19** | 77 | F | Hemorrhage | ICH | Lt. BG, thalamus | Lt. | 4 |
| **20** | 77 | F | Infarction | Infarction with HT | Lt. BG infarction | Lt. | 8 |

Abbreviations: DoC, Disorders of consciousness; Rt, Right; Lt, Left; M, Male; F, Female; Hypoxic, hypoxic brain injury; DAI, Diffuse axonal injury; ICH, Intracerebral hemorrhage; IVH, Intraventricular hemorrhage; HT, Hemorrhagic transformation; SAH, Subarachnoid hemorrhage; BG, Basal ganglia; PCA, Posterior cerebral artery; DACA, Distal anterior cerebral artery; Pcom, Posterior communicating artery; CRS-R, Coma Recovery Scale-Revised.
